# Supplementary material for: Effect of nutrition survey ‘cleaning criteria’ on estimates of malnutrition prevalence and disease burden: secondary data analysis
Source: PeerJ. 2014 May 13;2:e380. doi: 10.7717/peerj.380 (PMC4034601; doi:10.7717/peerj.380)
Supplement: Table S1 [file peerj-02-380-s001.docx]

**Webappendix**

**Table S1:** Details regarding the DHS surveys used in the analysis, including: country, year of survey, sample size for children aged 6-59 months and estimated population of children aged 6-59 months.

| **Country** | **Year of survey** | **Sample size**  **(6-59 months)** | **Estimated population**  **(6-59 months)** |
| --- | --- | --- | --- |
| **Bangladesh** | 2004 | 5,979 | 15,659,000 |
| **Burkina Faso** | 2003 | 8,278 | 2,213,000 |
| **Cambodia** | 2005 | 3,344 | 1,652,000 |
| **Cameroon** | 2004 | 3,189 | 2,208,000 |
| **Cote D'Ivoire** | 1998/9 | 1,710 | 2,496,000 |
| **Egypt** | 2005 | 12,202 | 8,040,000 |
| **Ethiopia** | 2005 | 4,368 | 11,757,000 |
| **Ghana** | 2003 | 2,928 | 2,792,000 |
| **Guatemala** | 1998/9 | 3,608 | 1,818,000 |
| **India** | 2005/6 | 45,398 | 108,010,000 |
| **Kenya** | 2003 | 4,910 | 5,162,000 |
| **Madagascar** | 2003/4 | 5,072 | 2,795,000 |
| **Malawi** | 2004 | 7,835 | 2,106,000 |
| **Mali** | 2006 | 11,005 | 2,342,000 |
| **Mozambique** | 2003/4 | 8,236 | 2,962,000 |
| **Niger** | 2006 | 3,976 | 2,566,000 |
| **Nigeria** | 2003 | 4,516 | 20,031,000 |
| **Peru** | 2000 | 10,507 | 2,697,000 |
| **Tanzania** | 2004 | 7,268 | 5,441,000 |
| **Turkey** | 2003 | 3,672 | 6,491,000 |
| **Zambia** | 2001/2 | 5,227 | 1,810,000 |
