# Supplementary material for: Effect of nutrition survey ‘cleaning criteria’ on estimates of malnutrition prevalence and disease burden: secondary data analysis
Source: PeerJ. 2014 May 13;2:e380. doi: 10.7717/peerj.380 (PMC4034601; doi:10.7717/peerj.380)
Supplement: Table S2 [file peerj-02-380-s002.docx]

**Webappendix**

**Table S2:** Estimated clinical caseloads for SAM in each country under different cleaning criteria.

| **Country** | **Estimated SAM caseload by cleaning criteria** | | | | | |
| --- | --- | --- | --- | --- | --- | --- |
|  | **No cleaning criteria applied** | **WHO 2006 Growth Standards** | **SMART flags** | **WHO 1995 Flexible criteria** | **WHO 1995 Fixed criteria** | **Epi-Info** |
| **India** | *10,408,000* | 8,684,000 | 6,476,000 | 8,769,000 | 6,270,000 | 6,093,000 |
| **Nigeria** | *1,683,000* | 1,181,000 | 312,000 | 913,000 | 819,000 | 766,000 |
| **Ethiopia** | *917,000* | 676,000 | 274,000 | 595,000 | 437,000 | 398,000 |
| **Bangladesh** | *770,000* | 663,000 | 534,000 | 665,000 | 530,000 | 512,000 |
| **Burkina Faso** | *344,000* | 265,000 | 154,000 | 251,000 | 178,000 | 175,000 |
| **Madagascar** | *240,000* | 204,000 | 93,000 | 192,000 | 131,000 | 125,000 |
| **Egypt** | *236,000* | 196,000 | 0 | 51,000 | 117,000 | 93,000 |
| **Mali** | *230,000* | 181,000 | 94,000 | 169,000 | 123,000 | 118,000 |
| **Kenya** | *200,000* | 153,000 | 12,000 | 111,000 | 102,000 | 99,000 |
| **Niger** | *180,000* | 140,000 | 81,000 | 128,000 | 104,000 | 101,000 |
| **Ghana** | *124,000* | 91,000 | 26,000 | 64,000 | 61,000 | 60,000 |
| **Mozambique** | *113,000* | 82,000 | 0 | 54,000 | 54,000 | 52,000 |
| **Malawi** | *111,000* | 85,000 | 0 | 39,000 | 59,000 | 51,000 |
| **Tanzania** | *85,000* | 65,000 | 0 | 51,000 | 51,000 | 47,000 |
| **Cameroon** | *78,000* | 65,000 | 0 | 44,000 | 50,000 | 45,000 |
| **Cote D'Ivoire** | *73,000* | 66,000 | 18,000 | 50,000 | 43,000 | 41,000 |
| **Zambia** | *63,000* | 52,000 | 0 | 37,000 | 38,000 | 33,000 |
| **Guatemala** | *45,000* | 33,000 | 0 | 17,000 | 22,000 | 22,000 |
| **Cambodia** | *33,000* | 28,000 | 14,000 | 27,000 | 21,000 | 21,000 |
| **Turkey** | *32,000* | 31,000 | 0 | 8,000 | 31,000 | 29,000 |
| **Peru** | *17,000* | 13,000 | 0 | 1,000 | 10,000 | 9,000 |
